# Annotations

**Published:** 1903-05-09

**Authors:** 


### Annotations.

### The London Hospital.

The Lord Mayor deserves hearty recognition for his hospitality to the governors, staff, and supporters of the London Hospital. It was an interesting gathering at the Mansion House on Monday evening, when the Duke of Cambridge, with his rugged sincerity and robust faith in the London Hospital, of which he has been president for more than half a century, was one of the features of the evening. Sir Frederick Treves described the reconstruction of the London Hospital as a colossal task, and paid a well-deserved compliment to the house committee and its chairman, the Hon. Sydney Holland, whom he testified had always been most ready to meet every demand which the medical staff had made upon their generosity. Sir Frederick further expressed his conviction that a cure for cancer would one day be found by the patient work of one man at his own cost, who would then give it freely to the world. In this way science progressed, and the ills of humanity were lessened. He paid a high tribute to Lord Lister, whose work had made hospitals popular with all classes and added immeasurably to the prolongation of life and the working powers of men and women. He further made the excellent point, that hospitals are essential to the successful treatment of disease in its worst forms, quite irrespective of the social position of the patient. The true meaning of the word hospital is a guest-house, and the English nation clung to this idea with a tenacity which did them credit, for it enabled the thoughtful of all classes in the days of health, by their money gifts, to entertain right royally the sick and suffering, whereby health was promoted and the powers of the nation were conserved. It is remarkable but true, as Mr. Sydney Holland pointed out, that several of our most famous hospitals owe most of their prosperity to the generous and substantial gifts of one or two individuals. The London Hospital, ministering as it did to the poorest members of the population, in one of the most crowded and humble districts of the metropolis, had never yet secured the whole individual interest of one wealthy man, who might make his name memorable for all time by coming forward at this period of its history and giving liberally, as Guy and Gassiot, Lords Mountstephen and Strathcona have given to great hospitals in the past. We would invite those who have large means to pay a visit to the London Hospital, because we are confident that the more they study its work and requirements, the more they will be attracted to the support of that institution. Then they may follow the example of the men who will go down to history as among the greatest benefactors of their day and generation.

### Milk Reform.

The last Amendment of the Food and Drugs Act made the addition of any preservative to milk illegal, but there still remain a good many points to thresh out before the question of what is, or is not, desirable treatment of milk intended for the nutrition of young children, can be regarded as settled. We have "sterilised milk" and "pasteurised milk," but the precise processes implied by these and many other terms vary with nearly every person who employs any one of them. Medical men are constantly being asked by parents which is the more desirable process, and if they are not more frequently consulted also as to whether milk should be boiled, it is because nowadays nearly every careful housewife takes it for granted that ordinary milk bought in an open vessel should be boiled before use irrespective of any process to which it may have been submitted previously. Each and every milk procedure has in its turn been accused of giving rise to infantile scurvy, anæmia, and rickets. It is quite certain however that no process will make milk from unhealthy cows healthy food for the human young, or restore to milk, originally of good character, those qualities which it has lost by being stored under improper conditions. All methods of milk treatment are but a qualified evil, and none of them would be necessary if municipal and other authorities did what they ought to. This is either to undertake the whole process of milk distribution themselves, or submit it, as a private trade, to much more stringent regulations than at present exist.

### Crime and Cremation.

The result of the Home Office inquiry into the question of cremation is a draft of regulations issued on the committee's report. The real difficulty is that the destruction of a body renders any further identification impossible, and the report of the committee recommends the most stringent formalities, one of which is a double certificate by the ordinary medical attendant and also by the official doctor. This seems a practical and reasonable safeguard against the concealment of crimes, such, for example, as those committed by Klosowski alias Chapman, who, if he had cremated his victims, would in all probability have escaped conviction or even suspicion. Post-mortem precautions of the strictest kind are, in short, always necessary, as carelessness in this respect favours the criminal. And yet students of medical jurisprudence can recall some curious instances of laxity even in post-mortem examinations. For example, there was the case of J. P. Cook, who was poisoned at Rugeley in 1856 by the notorious Dr. Palmer. No strychnine was found in the victim's stomach because it had been cut from end to end by Palmer himself during the post-mortem, at which the already suspected poisoner was actually allowed to assist! In another case, at Paris, the suspected murderer was permitted by the authorities to be present at the post-mortem, with the result that one of the jars—and that the most important one—sealed up for analysis was surreptitiously removed, and in the absence of all direct evidence of foul play the insurance companies had to pay heavy sums to an individual who there were strong grounds for believing to be a poisoner of exceptional skill and cunning. That a general medical practitioner who has not given special attention to toxicology may make the blunder of giving an erroneous certificate is obvious. When Miss Abercrombie was killed by a dose of strychnine administered by the infamous literary scoundrel Wainewright in 1830, her death was medically attributed to convulsions produced by eating oysters! An error of this sort prior to the body of the victim being cremated is all that a poisoner can wish for.

